# Phase I/II Study of Docetaxel and S-1 in Previously-Treated Patients with Advanced Non-Small Cell Lung Cancer: LOGIK0408

**DOI:** 10.3390/jcm8122196

**Published:** 2019-12-12

**Authors:** Koichi Takayama, Junji Uchino, Masaki Fujita, Shoji Tokunaga, Tomotoshi Imanaga, Ryotaro Morinaga, Noriyuki Ebi, Sho Saeki, Kazuya Matsukizono, Hiroshi Wataya, Tadaaki Yamada, Yoichi Nakanishi

**Affiliations:** 1 Research Institute for Diseases of the Chest, Graduate School of Medical Sciences, Kyushu University, Fukuoka 8190395, Japan; takayama@koto.kpu-m.ac.jp (K.T.); naka24@e-mail.jp (Y.N.); 2 Department of Respiratory Medicine, Kyoto Prefectural University of Medicine, Kyoto 6020841, Japan; tayamada@koto.kpu-m.ac.jp; 3 Department of Respiratory Medicine, Fukuoka University Hospital, Fukuoka 8140133, Japan; mfujita@fukuoka-u.ac.jp; 4 Medical Information Center, Kyushu University Hospital, Fukuoka 8190395, Japan; toksan@med.kyushu-u.ac.jp; 5 Department of respiratory disease, Nippon Steel Yawata Memorial Hospital, Kitakyushu 8058508, Japan; imanaga.t@ns.yawata-mhp.or.jp; 6 Department of Medical Oncology, Oita University Faculty of Medicine, Yuhu 8795593, Japan; r-morinaga@oitapref-hosp.jp; 7 Department of Respiratory Medicine, Iizuka Hospital, Iizuka, 8208505 Japan; nebi1@aih-net.com; 8 Department of Respiratory Medicine, Kumamoto University Hospital, Kumamoto 8608556, Japan; saeshow@wg7.so-net.ne.jp; 9 Department of Internal Medicine, Kagoshima City Hospital, Kagoshima 8908544, Japan; kmatsukiz@yahoo.co.jp; 10 Department of Internal Medicine, Saiseikai Fukuoka General Hospital, Fukuoka 8100001, Japan; h-wataya@saiseikai-hp.chuo.fukuoka.jp

**Keywords:** non-small cell lung cancer, previously treated patients, phase I/II trial, chemotherapy, docetaxel, S-1

## Abstract

Background: As docetaxel plus S-1 may be feasible for cancer treatment, we conducted a phase I/II trial to determine the recommended docetaxel dose and the fixed S-1 dose (phase I), as well as confirm the regimen’s efficacy and safety (phase II) for previously-treated patients with advanced non-small cell lung cancer. Methods: Patients ≤75 years with performance status ≤1 and adequate organ function were treated at three-week intervals with docetaxel on day 1 and 80 mg/m^2^ oral S-1 from days 1–14. The starting docetaxel dose was 45 mg/m^2^ and this was escalated to a maximum of 70 mg/m^2^. In phase II, response rate, progression-free survival (PFS), overall survival (OS), and safety were assessed. Results: The recommended doses were 50 mg/m^2^ docetaxel (day 1) and 80 mg/m^2^ S-1 (days 1–14). Grades 3 and 4 leukocytopenia and neutropenia occurred in 44% and 67% of patients, respectively. Nonhematologic toxicities were generally mild. Overall response to chemotherapy was 7.7% (95% confidence interval (CI), 1.6–20.9%), and median PFS and OS were 18.0 weeks (95% CI; 11.3–22.9 weeks) and 53.0 weeks, respectively. Conclusion: Fifty mg/m^2^ docetaxel plus 80 mg/m^2^ oral S-1 had a lower response rate than anticipated; however, the survival data were encouraging. A further investigation is warranted to select the optimal patient population.

## 1. Introduction

Previous clinical trials confirmed that docetaxel alone displays good anticancer effects when used to treat non-small cell lung cancer. Treatment with docetaxel is associated with significant prolongation of survival [1]. Docetaxel is an antineoplastic taxoid prepared by partial chemical modification of the non-cytotoxic precursor 10-deacetyl baccatin III, which is extracted from the needles of the European pine. Its mechanism of action is to promote the polymerization and depolymerization of microtubule proteins, resulting in microtubule stabilization and microtubule hyperplasia, which prevent chromosome migration and arrest cell division in the M phase of the cell cycle [2]. Docetaxel is useful as a second-line chemotherapy for patients that are refractory to conventional platinum-based chemotherapy [3]. When combined with platinum, docetaxel displays better responses and survival rates than other platinum-containing regimens [4,5]. S-1 is a novel oral anticancer drug composed of the 5-fluorouracil (5-FU) prodrug, tegafur, and two 5-FU modulators, 5-chloro-2,4-dihydroxypyridine (CDHP) and potassium oxonate. CDHP selectively antagonizes the rate-limiting enzyme dihydropyrimidine dehydrogenase in the 5-FU degradation pathway and enhances antitumor effects by increasing blood 5-FU levels. Potassium oxonate also selectively antagonizes orotate phosphoribosyltransferase in the gastrointestinal tract after oral administration and inhibits the formation of 5-fluoronucleotides from 5-FU. In a previous phase II study, monotherapy with S-1 produced a significant response in previously treated non-small cell lung cancer [6]. Because docetaxel and S-1 have different mechanisms of action and toxicity profiles, it may be feasible and efficient to combine these drugs for the treatment of advanced non-small cell lung cancer. Previously, Yoshida et al. reported the feasibility and usefulness of this combination chemotherapy in a clinical trial for advanced gastric cancer [7].

To improve the response rate in previously-treated patients with advanced non-small cell lung cancer, we conducted a phase I/II clinical study of docetaxel plus S-1. In this regimen, docetaxel was administered on day 1 while S-1 was administered on days 1 to 14, according to the potential schedule dependency previously reported by Kano et al. [8]. The primary objectives were to determine the maximum-tolerated dose (MTD) and the recommended dose for this regimen in a phase I study and confirm its efficacy and safety in the phase II study.

## 2. Experimental Section

### 2.1. Eligibility

Patients were enrolled in this study if they met the following eligibility criteria: cytologically or histologically confirmed diagnosis of incurable, previously treated non-small cell lung cancer; no previous use of docetaxel or uracil plus tegafur (UFT); age between 20–75 years; performance status of ≤1 on the Eastern Cooperative Oncology Group (ECOG) scale; an estimated life expectancy of >12 weeks; adequate bone marrow function (leukocyte count ≥4000/µL, platelet count ≥100,000/µL, and hemoglobin level ≥9.5 g/dL); adequate hepatic function (bilirubin level ≤1.5 mg/dL and a serum ratio of aspartate amino transferase to alanine amino transferase (AST/ALT) ≤2.5 × UNL); adequate renal function (serum creatinine ≤1.5 mg/dL); a measurable lesion according to the Response Evaluation Criteria in Solid Tumors (RECIST) guidelines version 1.0; and provision of written informed consent. The exclusion criteria were: active infection, massive ascites or pleural effusion, symptomatic brain metastasis, uncontrollable diabetes mellitus, or severe comorbidity such as heart disease or renal disease, interstitial pneumonia, watery diarrhea, active concomitant malignancy, pregnancy or lactation, or other medical problems that could prevent compliance with the protocol. The following conditions were necessary: an interval of at least 4 weeks after the end of final therapy and recovery from the previous treatment.

### 2.2. Treatment and Dose Escalation Schedules in the Phase I Study

On day 1 of each cycle, docetaxel (Sanofi-aventis K.K., Tokyo, Japan) diluted with 500 mL of normal saline was administered as a 90-min infusion. S-1 (Taiho Pharmaceutical Company, Tokyo, Japan) was orally administered twice daily on days 1–14. The dose of S-1 was determined according to the patient’s body surface area as follows: <1.25 m^2^, 40 mg; 1.25–1.50 m^2^, 50 mg; and >1.5 m^2^, 60 mg. Combination chemotherapy was repeated every 3 weeks until progressive disease (PD) occurred.

In the phase I study, the starting dose of docetaxel was 45 mg/m^2^ (level 1). Docetaxel dose escalation was performed as follows: in the patient cohorts containing at least 3 patients at each dose level, if none of the patients treated at a given dose level experienced dose limiting toxicity (DLT) as defined below, patients were entered at the next dose level (50 mg/m^2^ at level 2, 60 mg/m^2^ at level 3, and 70 mg/m^2^ at level 4). If 1 or 2 of the 3 patients in the cohort experienced DLT, 3 additional patients were entered at the same level. MTD was then fixed and dose escalation was discontinued if all 3 patients in the 3-patient cohorts or ≥3 patients in the 6-patient cohorts experienced DLT. Adverse events were assessed in the first two cycles of the phase I study.

In the phase II study, the recommended dose of docetaxel determined in phase I was used in combination with S-1 in the same manner. The treatment regimen was repeated every 21 days until PD, patient withdrawal, or the occurrence of a serious adverse event. Granulocyte colony-stimulating factor (G-CSF) could be administered when either fever ≥38 °C with grade 3 or 4 neutropenia occurred. DLT was defined as: grade 4 neutropenia lasting for ≥4 days, grade 3 febrile neutropenia lasting ≥72 h, grade 3 thrombocytopenia, ≥grade 3 nonhematologic toxicity (besides nausea, vomiting, fatigue, and alopecia), ≥grade 2 interstitial pneumonia, or interruption of the S-1 medication for ≥7 days. If a patient experienced DLT, the docetaxel dose was reduced by one level in the subsequent cycle. To receive a subsequent cycle of chemotherapy, patients had to have leukocyte counts ≥3000/mm^3^, neutrophil counts ≥1500/mm^3^, platelets ≥100,000/mm^3^, serum creatinine <1.5 mg/dL, and reduction in any treatment-related nonhematologic toxicity to <grade 1 (besides alopecia and neuropathy).

### 2.3. Toxicity and Response Evaluation

Toxicity was evaluated according to the Common Terminology Criteria for Adverse Events, version 3.0. Patients’ symptoms and general condition were observed periodically. Physical examinations, complete blood counts with differential counts, serum chemistry, and urine tests were carried out at least once per week during the DLT-evaluation period. Tumor response was evaluated according to RECIST version 1.0 every month until the final tumor response was determined. Progression-free survival (PFS) was defined as the time from the date of registration to the date of the first documentation of PD or death. Patients with PFS were censored at the last date when survival was verified. Overall survival (OS) was defined as the time from the date of registration to the date of death. Surviving patients were censored at the last confirmation date of survival. This phase I/II study was conducted in accordance with the Declaration of Helsinki and approved by the Institutional Review Board at each participating hospital. The study was monitored by an independent data and safety monitoring committee.

### 2.4. Statistical Considerations

The primary end point of the phase II study was the rate of response to combination chemotherapy. The study was powered to detect a significant improvement of 18% relative to the 5% estimated from previous studies [1,3]. Assuming a one-sided a = 0.05% and 80% power, sample size was calculated to be 39 patients, with 6 patients at the recommended dose level in the phase I study. PFS and OS were assessed by the Kaplan-Meier method and log-rank test.

## 3. Results

### 3.1. Phase I Study

In total, 12 patients were enrolled in the phase I study. None of the three patients in the cohort at level 1 developed DLT. When the docetaxel dose was elevated to level 2, one patient experienced DLT, a grade 3 nonhematologic toxicity. The patient had phenytoin intoxication, which might be due to impairment of the P450 metabolic pathway by S-1. Because the remaining three patients in the cohort at level 2 had no DLT, their docetaxel dose was elevated. At level 3, two patients had DLT, prolonged grade 3 myelosuppression, and grade 3 interstitial pneumonia. The safety committee emphasized the risk of interstitial pneumonia and recommended the termination of the phase I study at level 3. As a result, the MTD and recommended docetaxel dose were 60 mg/m^2^ and 50 mg/m^2^, respectively.

### 3.2. Patient Characteristics in Phase II Study

A total of 39 patients (31 men, 8 women; median patient age, 64 years; age range, 46 to 75 years) were enrolled in the phase II study. Patient characteristics are shown in Table 1. The Eastern Cooperative Oncology Group (ECOG) performance status was 0 for 18 patients and 1 for 21 patients. Histologically, there were 24 patients with adenocarcinoma, 11 with squamous cell carcinoma, and 4 with no specified histology. Twenty-nine and 10 patients were at the clinical stages of IV and IIIb, respectively. A total of 120 cycles of therapy were administered. Treatment delay or interruption in the administration of S-1 occurred in 27 cycles (23%). The median number of cycles administered per patient was 3 (range, 1–9). The relative dose intensities were 97.7% for docetaxel and 85.7% for S-1.

### 3.3. Toxicity

All 39 patients were included in the safety assessment. As shown in Table 2, myelosuppression was the principal toxic effect observed. However, the degree of myelosuppression was generally mild. Grade 3 or 4 leukocytopenia, neutropenia, thrombocytopenia, and anemia occurred in 17 (44%), 26 (67%), 0 (0%), and 0 (0%) patients, respectively. Nonhematologic toxicity was also generally mild and less frequent. Grade 3 or 4 toxicity primarily included loss of appetite (7.7%), fever (5.1%), and interstitial pneumonia (5.1%).

### 3.4. Efficacy

**Figure 1:**
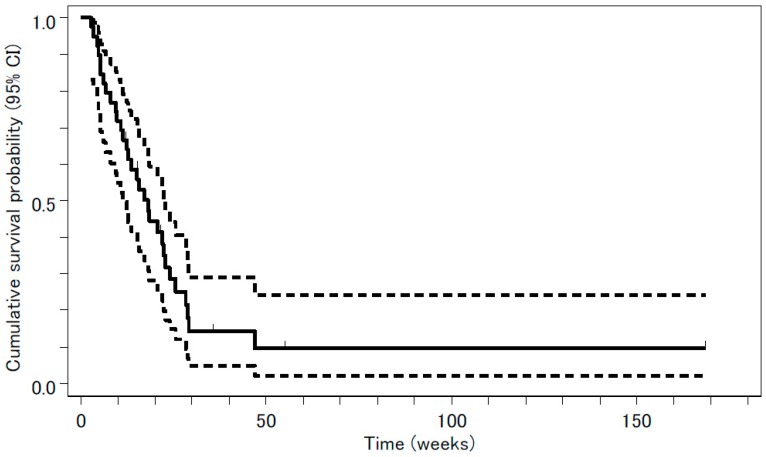
Progression-free survival (PFS) analyzed by the Kaplan-Meyer method is presented as a solid line. Median PFS was 18.0 weeks (95% confidence interval (CI), 11.3–22.9 weeks). The 95% CI is presented as two dashed lines.

**Figure 2:**
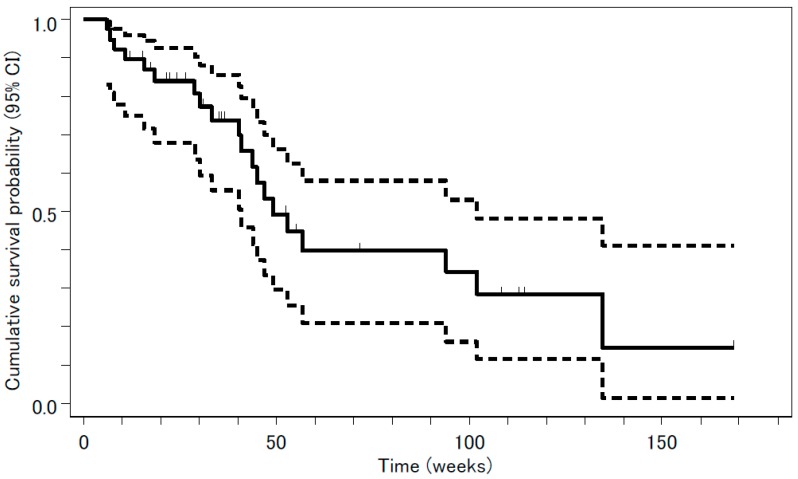
Overall survival (OS) analyzed by the Kaplan-Meyer method is presented as a solid line. Median OS was 53.0 weeks (95% confidence interval (CI), 40.9–134.6 weeks). The 95% CI is presented as two dashed lines.

Tumor response was evaluated in 39 patients. The median follow-up period was 8 months (range, 1–39 months). The following results were found for treatment response: complete response (CR), 0; partial response (PR), 3; stable disease (SD), 24; PD, 5; and not evaluable (NE), 7. The overall response rate was 3/39 or 7.7% (95% confidence interval (CI), 1.6 –20.9%, *p* = 0.31), a value that was not significantly higher than the threshold response rate statistically estimated from previous studies. The rate of disease control, CR + PR + SD, was 27/39 or 69%. Median PFS and OS were 18.0 weeks (95% CI, 11.3–22.9 weeks) and 53.0 weeks (95% CI, 40.9–134.6 weeks), respectively. The survival curves are shown in Figure 1 and Figure 2.

## 4. Discussion

In the present study, response rate did not meet the 18% criterion needed to establish a significant improvement relative to the previously reported rate (i.e., the primary endpoint for the efficacy in the phase II study). However, combination chemotherapy was well tolerated and resulted in encouraging survival data.

Based on large-scale randomized controlled trials that compared 75 mg/m^2^ docetaxel to optimal supportive care or other anticancer drugs, docetaxel monotherapy is considered to be a standard treatment for advanced non-small cell lung cancer in second-line settings [1,3]. Furthermore, S-1 was confirmed to be effective for previously treated advanced non-small cell lung cancer. Evidence for the efficacy of combination chemotherapy with docetaxel and S-1 has mainly been derived from studies on advanced gastric cancer. S-1 is one of the preferred agents for the treatment of gastric cancer. In fact, docetaxel displayed synergism with S-1 in vitro, improving the response rates in several phase II trials for advanced gastric cancer [7,9,10,11]. Wada et al. reported the decrease in expression of thymidylate synthase (TS) and dihydropyrimidine dehydrogenase (DPD) and an increase in expression of orotate phosphoribosyl transferase (OPRT) after co-treatment with docetaxel plus FU compared to 5-FU alone in gastric cancer cell lines [9]. DPD catalyzes the metabolic inactivation of 5-FU, while OPRT directly converts 5-FU to 5-fluorouridine-5′-monophosphate (FUMP), an active metabolite that displays anticancer effects. These changes in enzymes that metabolize 5-FU might clarify the enhanced effect of S-1 combined with docetaxel. As reported by Hasegawa et al., a similar synergistic effect is observed in castration-resistant prostate cancer [12]. Interestingly, in the xenograft model, S-1 with low-dose docetaxel could enhance the antitumor effect of S-1.

Several clinical trials have been conducted using docetaxel and S-1 for chemotherapy-naive or previously treated non-small cell lung cancer [9,13,14,15,16,17]. In fact, a triweekly schedule was employed with 40 mg/m^2^ as the starting docetaxel dose. Previously, Oki et al. reported the usefulness of biweekly administration of docetaxel [16]. Table 3 contains a summary of results from these clinical studies. Other studies used a fixed docetaxel dose of 40 mg/m^2^ + S-1 80 mg/m^2^. The relatively low efficacy in these studies was perhaps due to the low fixed dose of chemotherapeutic agents. Therefore, we employed docetaxel dose escalation in the phase I part of the study, and confirmed that 50 mg/m^2^ of docetaxel was effective. Moreover, S-1 dosage was determined depending on body surface area (BSA) in each patient in this study, because the fixed dose of S-1 may be too toxic for patients with smaller BSA or ineffective for the patients with larger BSA. Although the response rate in the current study was relatively low compared to that in other studies, the disease control rate was better than those of other studies (69% in this study, 68.9% in Atagi et al., 61% in Segawa et al., 84% in Yanagihara et al., and 49% in Oki et al.) and favorable PFS and OS were obtained. In addition, the PFS was longer than that reported in a previous phase III study [18] where treatment efficacy was compared between docetaxel and gefitinib for previously treated patients with non-small cell lung cancer in Japan; the PFS in the arm treated with docetaxel was 2.0 months. For toxicity, we found that ≥grade 3 myelosuppression was more frequent in our study and may be due to the higher docetaxel dose employed relative to other studies. Nonetheless, nonhematologic toxicity was demonstrated to be mild, and 50 mg/m^2^ docetaxel plus S-1 was generally well tolerated.

Takeda et al. reported that in advanced non-small cell lung cancer, the expression of TS and DPD are associated with the response to S-1 and carboplatin [19]. At a low level, their expression was found to be associated with a better response and a longer survival in patients treated with S-1 and carboplatin. Altogether, the expression levels of TS and DPD may be predictive markers for the response to regimens containing S-1. Histologically, squamous cell carcinoma and high-grade carcinoma display higher expression levels for the TS protein and mRNA in non-small cell lung cancer [20,21]. Therefore, a selected patient population with lower expression levels of TS may be most suitable for treatment regimens containing TS-inhibiting agents such as the combination of docetaxel and S-1. We have a plan to conduct a study considering this in the future.

## Figures and Tables

**Table 1:** Characteristics of patients in the phase II study.

Characteristics	N = 39
Age, years	
Median (range)	64 (46–75)
Sex	
Men	31
Women	8
ECOG PS	
0	18
1	21
Stage	
IIIb	10
IV	29
Histology	
Adenocarcinoma	24
Squamous cell carcinoma	11
Not specified	4

ECOG PS: Eastern Cooperative Oncology Group performance status.

**Table 2:** Toxicities in the phase II study.

Toxicity	Grade		
	3	4	3 + 4
Hematologic			
Leukocytopenia	15 (38.5%)	2 (5.1%)	17 (43.6%)
Neutropenia	14 (35.9%)	12 (30.8%)	26 (66.7%)
Anemia	0 (0%)	0 (0%)	0 (0%)
Thrombocytopenia	0 (0%)	0 (0%)	0 (0%)
Nonhematologic			
Loss of appetite	3 (7.7%)	0 (0%)	3 (7.7%)
Fever	2 (5.1%)	0 (0%)	2 (5.1%)
Pneumonitis	1 (2.6%)	1 (2.6%)	2 (5.1%)
Stomatitis	1 (2.6%)	0 (0%)	1 (2.6%)
Diarrhea	1 (2.6%)	0 (0%)	1 (2.6%)
Hypercalcemia	1 (2.6%)	0 (0%)	1 (2.6%)
Elevation of γGTP	1 (2.6%)	0 (0%)	1 (2.6%)

**Table 3:** Summary of the results from previous trials of docetaxel plus S-1.

	Author			
	Atagi et al. [12]	Yanagihara et al. [14]	Segawa et al. [13]	Oki et al. [15]
N	29	28	31	49
Docetaxel	40 mg/m^2^, day 1 every 3 weeks	40 mg/m^2^, day 1 every 3 weeks	40 mg/m^2^, day 1 every 3 weeks	35 mg/m^2^, day 1, 15 every 4 weeks
S-1	80 mg/m^2^, days 1–14	80 mg/m^2^, days 1–14	80 mg/m^2^, days 1–15	80 mg/m^2^, days 1–14
ORR	24.1%	18.4%	16.1%	16.3%
PFS (mo)	3.9	4.4	3.4	3
OS (mo)	11.8	16.1	8.7	9

ORR: overall response rate; OS: overall survival; PFS: progression-free survival.
